# Psychological distress among healthcare professionals in Mbarara, following the 2022 Ebola Virus Disease outbreak in Uganda: a mixed methods study

**DOI:** 10.1186/s12888-024-05922-w

**Published:** 2024-06-25

**Authors:** Joan Abaatyo, Godfrey Zari Rukundo, Margaret Twine, Dan Lutasingwa, Alain Favina, Novatus Nyemara, Rosemary Ricciardelli

**Affiliations:** 1 Department of Psychiatry, Faculty of Medicine, Uganda Christian University, Kampala, Uganda; 2 King Ceasor University, Kampala, Uganda; 3 Department of Psychiatry, Faculty of Medicine, Mbarara University of Science and Technology, Mbarara, Uganda; 4 Health Development Initiative, Kigali, Rwanda; 5 Fisheries and Marine Institute, Memorial University of Newfoundland, St. John’s, Canada

**Keywords:** Psychological distress, Health care professionals, Ebola virus disease

## Abstract

**Background:**

The 2022 Ebola Virus Disease (EVD) outbreak occurred at a time when Uganda was still battling the social and psychological challenges of the COVID-19 pandemic; placing health care professionals (HCPs) at a much higher risk of developing psychological distress. Psychological distress among HCPs can cause decreased workplace productivity and ineffective management of their patients. The current study aimed to investigate and understand psychological distress among HCPS in Mbarara city in Southwestern Uganda following the 2022 EVD outbreak.

**Method:**

We enrolled 200 HCPs through convenient sampling from one private and one public health facility in Mbarara city in Southwestern Uganda, in a cross-sectional convergent parallel mixed method approach where qualitative and quantitative data were collected concurrently. Quantitative data, utilizing the Kessler Psychological Distress (K10) Scale, provided us with a quantitative measure of the prevalence of psychological distress among HCPs, and were analyzed using STATA version 16. Qualitative data, on the other hand, offered deeper insights into the nature, perceptions, and contextual factors influencing this distress, and were analyzed using emergent theme analysis.

**Results:**

The prevalence of psychological distress was 59.5% and it was higher among females (63.9%) compared to males (36.1%). HCPs vividly expressed distress and anxiety, with heightened suspicion that every patient might be an EVD carrier, creating a pervasive sense of unsafety in the workplace. However, the outbreak had an educational affect where concerns about the announcement of another EVD outbreak were diverse, with HCPs expressing anxiety, despair, and dissatisfaction with the country’s management of potential outbreaks.

**Conclusion:**

High levels of psychological distress were experienced by HCPs in Southwestern Uganda as a result of the 2022 EVD pandemic. HCPs express a wide range of feelings, such as dread, anxiety, despair, pessimism, and discontent with the way the outbreaks are handled throughout the nation. We recommend implementation of comprehensive psychosocial support programs tailored to the unique needs of HCPs, including counseling services, stress management workshops, and peer support networks.

## Introduction

In September, 2022 an outbreak of Ebola Virus Disease (EVD) was announced in Mubende district located in central Uganda [1]. In total, this outbreak impacted nine districts: Bunyangabu, Jinja, Kagadi, Kampala, Kassanda, Kyegegwa, Masaka, Mubende, and Wakiso [2]. During the outbreak, 142 confirmed cases of EVD caused by Sudan Ebola virus including 55 deaths were reported [2]. Among the confirmed cases were 19 healthcare professionals (HCPs), most of whom were medical doctors, who accounted for 7 of the deaths caused by the virus [2]. An additional 22 probable cases died before samples were obtained for confirmation and there were 87 recoveries among the confirmed cases [1].

Sudan Ebola is one of the three variants of Ebola viruses that cause hemorrhagic fever, which was first discovered in 1979 in southern Sudan [3]. The Zaire Ebola was discovered near the Ebola River in the Democratic Republic of Congo [4]. Bundibugyo ebolavirus (BEBV) was first discovered in the Bundibugyo District of Uganda in 2007 during an outbreak of EVD [5]. The Sudan Ebola virus is highly infectious and fatal [6, 7]. The risk of infection among HCP is heightened by the fact that Ebola initially presents with non-specific symptoms like fever, headache, muscle pain and chills, contrary to the most feared presentation of bleeding from orifices which happens in the late stages of the disease [6, 7]. EVD spreads through contact with infected bodily fluids to which the HCPs are exposed since they usually have to physically examine the patients without appropriate personal protective equipment (PPE) [8]. There is scarcity of PPE in healthcare settings in low-income countries and HCPs often examine patients without being protected [9].

In acknowledging initial symptoms of EVD are non-specific, during an out-break, HCPs in high-risk healthcare facilities or areas are likely to treat every patient as a potential carrier [10], which can undermine their quality of care and be demotivating. In addition, the anticipation of every patient having EVD disease, suggests HCPs to live in fear and anxiety which may escalate into psychological distress [11]. Moreover, provision of medical care for patients with EVD is generally associated with physical and psychological distress due to extended shift times, the long process of donning and doffing, and the rigorous monitoring of the critically ill patients [12]. Due to increased exposure via occupational responsibilities, HCPs are more likely to be infected than the general adult population, and psychological distress from exposure to death with uncertainty of contracting the disease further increases distress among HCPs [13]. A study done in Sierra-Leonne showed a prevalence of anxiety of 48% among HCPs in Ebola pandemic, and perceived Ebola threat was the main predictor for anxiety among the study participants [14]. Other studies done in West Africa have reported that, yet HCPs are more likely to get firsthand information about people in isolation [15–17]. Psychological distress may vary among HCPs across the country, with those directly caring for EVD patients facing the highest risk. In contrast, HCPs in routine care or continuity, especially in areas where EVD has not yet occurred, may experience comparatively lower levels of psychological distress.

Anxiety from psychological distress hinders one’s working memory, causing forgetfulness of essential tasks for HCPs [18]. Forgetfulness can impair HCPs work performance and reduce safety at work as they could forget the standard operating procedures to alleviate disease spread [18, 19]. Psychological distress endangers HCPs and poses risks to their colleagues, families, and the general public [19]. Furthermore, psychological distress can elevate concerns regarding trust and transparency at work as well as decreased workplace productivity and ineffective management of their patients [20, 21]. In addition, psychological distress among HCPs can cause emergence of other psychiatric disorders symptoms, like those of paranoia and depression [22]. Psychological distress among HCPs may influence selection into other less advantaged occupations with poorer working conditions, that may increase the risk of future depressive disorders [23].

Uganda has thus far had five EVD out breaks majorly affecting the western and central regions of the country [24], and the most recent outbreak started in September 2022 [1]. Most outbreaks predominantly featured the Sudan Ebola virus variant, except for the 2007 Bundibugyo outbreak, attributed to the Bundibugyo ebolavirus (BEBV) which also accounted for the most limited deaths [25]. Geographically, they were largely concentrated in rural areas of Uganda, often affecting single districts [25]. However, the 2022 outbreak expanded across multiple districts, extending into both rural and urban settings [2]. Like the initial outbreak, a considerable number of healthcare professionals were engaged in the 2022 event [2, 26].

Psychological distress resulting from the issues surrounding EVD such as scarcity of personal protective equipment (PPE), non-specific initial EVD symptoms that most patients present with as possible carriers, fear of contracting the disease, and the risk of dying once one contracts the disease; impair work productivity of HCPs who pose a danger to themselves, their colleagues, family, and the general public. Resultantly, HCPs are predisposed to other mental health challenges in the future [27]. Our study is crucial due to the lack of available data on the psychological distress experienced by HCPs in Uganda following EVD outbreaks, despite the country’s history of such outbreaks. Additionally, we sought to reveal the extent of the psychological impact on HCPs in Uganda, especially considering the timing of the recent Ebola outbreak alongside the ongoing COVID-19 pandemic. This confluence of infectious disease outbreaks likely exacerbated the psychological burden on HCPs, who were already under immense pressure and at heightened risk of distress [28].

Exploring the psychological distress among HCPs in Mbarara in Southwestern Uganda following the 2022 EVD outbreak offers valuable insights into the broader context of healthcare worker well-being and resilience. By understanding the specific challenges faced by HCPs in this region, policymakers and healthcare organizations can tailor support systems and interventions to address their unique needs. Furthermore, this study contributes to the broader understanding of the impact of infectious disease outbreaks on healthcare systems and the value of prioritizing the mental health of frontline workers in epidemic response efforts. Thus, we sought to investigate and understand psychological distress among HCPS in Mbarara city in Southwestern Uganda following the 2022 EVD outbreak.

## Methods

### Study Design

The current study, conducted between September to November, 2023, employed a cross-sectional convergent parallel mixed method approach where qualitative and quantitative data were collected concurrently, analyzed separately, and combined at discussion level to nuance the experience of psychological distress with the context underpinning the distress. Triangulation of the data occurred when discussing the integrated findings, which we synthesized to explore the nuanced relationship between psychological distress and its contextual factors, facilitating a deeper interpretation of the results and their implications.

### Study setting

We conducted the study at two health centers in Mbarara city in southwestern Uganda: one private and one public hospital, specifically Mbarara Regional Referral Hospital (MRRH) (public) and Divine Mercy Hospital (one of the largest private hospitals in Mbarara). While Mbarara City has 19 health facilities, most of which are private, only MRRH was under high alert to receive patients with EVD because it is a regional referral hospital for the western region.

Situated approximately 266 km southwest of Kampala, the capital city of Uganda, Mbarara is strategically positioned within proximity to several districts that have been affected by Ebola Virus Disease (EVD) outbreaks like Kampala, Kyegegwa, Masaka, and Mubende. The geographic proximity underscored the relevance of studying psychological distress among HCPs in Mbarara following the recent EVD outbreak. Understanding the experiences of HCPs in this region, which is close to areas affected by EVD, provides valuable insights into the broader context of epidemic response and healthcare worker well-being in Uganda.

### Study population and eligibility screening

Our study population included doctors, nurses, midwives, laboratory technicians, and clinical officers in Mbarara city of Uganda. We targeted HCPs because HCPs are the first line of contact for patients. While previous epidemics have shown that all HCPs are at high risk of EVD including radiographers, pharmacists, physiotherapists, optometrists, the choice of HCPs in this study we focused on those working on the frontline with patients. We excluded individuals who; (i) are too physically sick to withstand the length of the interview, (ii) had not been working at the study sites during the EVD outbreak i.e.; between September, 2022 to January, 2023. Convenient sampling was employed for both the study sites and study participants. MRRH has had a total of about 358 HCPs while Divine Mercy Hospital had about 45 HCPs, during the study period.

### Sample size estimation

The sample size was calculated using Kish Leslie’s formula [29] for a finite population. We followed the standard normal deviation, typically set at 1.96 for maximum sample size at a 95% confidence interval. With a constant probability (p) of 50% or 0.5 (as no measures were estimated), we calculated the complement (Q) as 1-p, resulting in Q = 0.5. The desired degree of accuracy (e) was set at 0.05 or a 0.05 probability level (at a 95% confidence level). Considering a 10% non-response rate, we arrived at a sample size of 213 which was allocated between the hospitals at a ratio of 4:1 for MRRH and Divine Mercy Hospital, respectively. However, 13 participants did not complete the questionnaire and were excluded from the analysis. Each cadre of HCPs was then sampled in proportion to its representation in the at study sites. The same number of participants (200) were involved in qualitative data collection. Our approach aimed to capture comprehensive insights from a diverse range of perspectives, ensuring richness in data and theoretical saturation [30].

### Data collection

All information was collected by four research assistants (RAs), each trained in data collection, research ethics, administration of questionnaires, and how to ask sensitive questions. RAs identified eligible participants who presented in the hospital, approaching each for an interview, where the RA led the survey completion, to be conducted in a private office or during unit/clinical meetings. Only after potential participants consented, would the RA hand them the survey instrument. To reduce the institutional footprint and care provision, RAs allocated time (during break of post shift) at their convenience to support participants in completing the questionnaire. While quantitative data, using the Kessler Psychological Distress (K10) Scale, provided us with a quantitative measure of the prevalence of psychological distress among HCPs, qualitative data offered deeper insights into the nature, perceptions, and contextual factors influencing this distress. The integration of qualitative and quantitative data allowed for a comprehensive examination of psychological distress among HCPs, providing both breadth and depth to our findings.

### Study variables

Our research explored numerous variables, encompassing both independent and dependent factors. Independent variables comprised Age, Health Profession (segmenting participants into four categories: Clinical Officers, Laboratory Technicians, Medical Doctors, and Nurses/Midwives), Gender, Marital Status, and Hospital Type (categorizing the workplace setting in terms of private and public). Our selection of sociodemographic variables was motivated by their recognized associations with psychological distress among healthcare professionals (HCPs) in previous research [31–35]. The Dependent Variable centered on Psychological Distress experienced by healthcare professionals, serving as the principal focus of our investigation.

### Study tools and measures

#### Quantitative data

The final, largely validated, survey included socio-demographic information such as age, sex, marital status, health profession and psychological distress which was assessed using the Kessler Psychological Distress Scale (K10) [36]. Qualitative questions were included in a questionnaire alongside quantitative questions.

### Study tools

The 10-item Kessler Psychological Distress Scale (K10), is a well-validated self-report tool for assessing psychological symptoms [36, 37]. The K10 been used by researchers in the Ugandan setting across various populations including HCPs [38], particularly in the context of infectious disease outbreaks and high-stress environments, refugee and displaced populations [39], HIV/AIDS affected populations [40] and adolescents [40]. It is composed of 10 questions with 5-item Likert-like responses i.e., none of the time, a little of the time, some of the time, most of the time, and all of the time. The responses are scored from 1 to 5 respectively and the overall score summed for each participant ranges from 10 to 50. Participants with a score of > 20 are considered to have PD. K10 has satisfactory psychometric properties and is strongly associated with the presence of posttraumatic stress disorder, major depressive disorder, generalized anxiety disorder, and panic disorder [41]. However, despite K10 being validated, we did modify all items to elicit responses relevant to the context of EVD outbreak. For instance, the original query “In the past 4 weeks, about how often did you feel tired out for no good reason?” was adjusted to “During the recent EVD outbreak, about how often did you feel tired out for no good reason?“. For this study the Cronbach alpha was 0.71.

### Qualitative data

We included qualitative questions within each questionnaire administered to all participants. Participants responded to open-ended survey items [42] in the questionnaire within the key domains of; (i) Their experience attending to general patients during the EVD outbreak. ii. how they would feel if another EVD outbreak was announced in their district of work. iii. the reasons (why) behind these feelings. We conducted a pretest with a subset of HCPs from each clinical practice cadre before including the final qualitative questions in the questionnaire to gather the qualitative data. Items would be asked in several ways until comprehension was evident if one of the HCPs taking the pretest did not understand the phrase. The collected data was inputted into a password-protected Microsoft Access database.

### Ethical considerations

We conducted the study following the guidelines of the Declaration of Helsinki 2013, with approval from the Research Ethics Committee of Mbarara University of Science and Technology (MUST-2023-779) and Uganda National Council for Science and Technology (HS3037ES). Written informed consent was obtained from participants prior to participation in the study.

### Data analysis

The quantitative data collected were cleaned and then analyzed using STATA version 16. We used descriptive statistics to summarize continuous variables (i.e., mean, mode, median, and standard deviations), while categorical variables were summarized using proportions and percentages. An independent sample t-test was utilized to compare numerical variables like age, and Pearson’s chi-square test was used to compare categorical data between the groups. We adjusted for hospital type clustering and employed Modified Poisson regression to examine how socio-demographic factors relate to psychological distress. All statistics were calculated at a 95% level of confidence and 5% statistical error.

For qualitative data, we completed an emergent theme analysis. Responses, alongside those from HCPs who piloted the survey, were analyzed such that themes were derived from the words of multiple participants who shared experiences [43]. A theme was constituted not only by multiple participants reporting on the same phenomenon in a consistent manner; but also recurring or cross-cutting pattern of responses. To this end, we made judgments when coding to decontextualize and recontextualize data [43]. In the quotes used, we stay verbatim, but protect the confidentiality and anonymity of the participants.

## Results

### Quantitative findings

A total of 200 HCPs participated in the study. Most of the participants (70.5%) were from the public hospital and most were nurses/midwives (54.0%) and majority were female (56.0). Additionally, most participants were married (52.0). The average age of the participants included was 32 ± 7.0 (See Table 1).

### **Prevalence of psychological distress following EVD outbreak and its distribution across study variables**

The prevalence of psychological distress was 59.5% (*n* = 119), 95% Confidence interval (CI) = 52.5 – 66.1%. Psychological distress was statistically more prevalent among females than males (63.9% vs. 36.1%, X^2^ = 7.38, p value = 0.007). There was no statistically significant difference in the prevalence of psychological distress between the public and private hospital. (See Table 1).

**Table 1:** Participant socio-demographic characteristics distribution across presence of psychological distress

Variable	*n* (%)	Psychological distress		X^2^ (*p*-value)
		No 81 (40.5)	Yes 119 (59.5) 95% CI (52.5–66.1)	
**Age** *[mean (SD)]*	32 (7.0)	32.3, 7.4	31.9, 6.8	0.717
**Health profession**				
Clinical officers	9 (4.6)	6 (7.4)	3 (2.5)	2.96 (0.399)
Laboratory technicians	16 (8.0)	6 (7.4)	10 (8.4)	
Medical doctors	67 (33.5)	28 (34.6)	39 (32.8)	
Nurses/midwives	108 (54.0)	41 (50.6)	67 (56.3)	
**Sex**				
Male	88 (44.0)	45 (55.6)	43 (36.1)	**7.38 (0.007)**
Female	112 (56.0)	36 (44.4)	76 (63.9)	
**Marital status**				
Single	96 (48.0)	41 (50.6)	55 (46.2)	0.38 (0.541)
Married	104 (52.0)	40 (49.4)	64 (53.8)	
**Hospital type**				
Private	41 (20.5)	13 (16.0)	28 (23.5)	1.65 (0.198)
Public	159 (70.5)	68 (84.0)	91 (76.5)	

### Participant responses to psychological distress items

Table 2 presents the frequency distribution of specific items describing psychological stress (PD) of our participants during the Ebola outbreak. All items indicated that the participants felt a lower to higher level of PD during the Ebola outbreak. However, “feeling nervous” is the first item with a higher percentage of participants (25.5%) suffering from PD all the time, while the second and the third items were respectively “feeling that everything is an effort” (11%) and “feeling tired out for no reason” (10%). Results showed that “feeling depressed” was the fourth item with 8% of the participants. “Feeling so nervous that nothing could calm you down” and “feeling so sad that nothing could cheer you up” are two items with an equal number of participants suffering from PD all the time (7%). Similarly, “feeling worthless” and “feeling so restless that you could not sit still” reported an equal number of the participants (5% for each item). “Feeling restless or fidgety” all time was felt by 6.5% of the participants, while “feeling hopeless” was felt by a less number of the participants (4.5%).

**Table 2:** Participant responses to items on the Kessler Psychological Distress Scale (K10)

K10 items	Responses *n* (%)				
	Never	A little of the time	some of the time	Most of the time	All the time
During the Ebola outbreak; how often did you feel tired out for no reason	44 (22.0)	34 (17.0)	63 (31.5)	39 (19.5)	20 (10.0)
During the Ebola outbreak; about how often did you feel nervous?	11 (5.5)	26 (13.0)	61 (30.5)	51 (25.5)	51 (25.5)
During the Ebola outbreak; about how often did you feel so nervous that nothing could calm you down?	63 (31.5)	41 (20.5)	50 (25.0)	32 (16.0)	14 (7.0)
During the Ebola outbreak; about how often did you feel hopeless?	76 (38.0)	46 (23.0)	47 (23.5)	22 (11.0)	9 (4.5)
During the Ebola outbreak; about how often did you feel restless or fidgety?	70 (35.0)	49 (24.5)	49 (24.5)	19 (9.5)	13 (6.5)
During the Ebola outbreak; about how often did you feel so restless you could not sit still?	100 (50.0)	46 (23.0)	28 (14.0)	16 (8.0)	10 (5.0)
During the Ebola outbreak; about how often did you feel depressed?	69 (34.5)	52 (26.0)	40 (20.0)	23 (11.5)	16 (8.0)
During the Ebola outbreak; about how often did you feel that everything was an effort?	42 (21.0)	49 (24.5)	49 (24.5)	38 (19.0)	22 (11.0)
During the Ebola outbreak; about how often did you feel so sad that nothing could cheer you up?	80 (40.0)	43 (21.5)	44 (22.0)	19 (9.5)	14 (7.0)
During the Ebola outbreak; about how often did you feel worthless?	114 (57.0)	34 (17.0)	32 (16.0)	10 (5.0)	10 (5.0)

### Factors associated with psychological distress

Being female compared to being male [adjusted prevalence ratio (aPR) = 1.40, CI = 1.21–1.63, p value = < 0.001], being a medical doctor [aPR = 1.72, CI = 1.19–2.51, p value = 0.004] and being a nurse/midwife compared to being a clinical officer [aPR = 1.61, CI = 1.11–2.23, p value = 0.012] increased the odds of psychological distress among HCPs (Table 3).

**Table 3:** Regression analysis for factors associated with psychological distress

Variable	Bi variable analysis		Multivariable analysis	
	Crude Prevalence ratio (95% confidence interval)	*p*-value	Adjusted Prevalence ratio (95% confidence interval)	*P*-value
**Age**	0.99 (0.98–1.00)	0.411	0.99 (0.99–1.00)	0.794
**Sex**				
Male	1 (reference)		1 (reference)	
Female	1.39 (1.17–1.65)	< 0.001	1.40 (1.21–1.63)	**< 0.001**
**Marital status**				
Single	1 (reference)		1 (reference)	
Married	1.62 (1.11–2.34)	0.011	1.02 (0.73–1.43)	0.870
**Health profession**				
Clinical officer	1 (reference)		1 (reference)	
Lab technician	1.88 (0.81–4.29)	0.137	1.88 (0.91–3.86)	0.086
Medical doctor	1.74 (1.17–2.61)	0.007	1.72 (1.19–2.51)	**0.004**
Nurse/midwife	1.86 (1.03–3.35)	0.039	1.61 (1.11–2.34)	**0.012**

### Qualitative findings

#### Experiences of HCPs during the EVD outbreak

The participants conveyed a great deal of distress and symptoms of anxiety, believing they could acquire EVD at any time while treating patients. Anxiety was exacerbated by a widespread suspicion of each patient as a potential EVD carrier. A persistent sensation of unsafety pervaded the work atmosphere, which was described as overpowering and unpleasant all the time. For example, a participant said: “I felt like I would contract EBV anytime” and another proclaimed “I was very scared of every patient in case they later turned out to be having EVD”. As the participants’ words, echoing others, evidence they took a cautious stance, avoiding physical contact and viewing every patient as contagious. As a precaution, efforts were made to always wear protective clothing (e.g., “I considered every patient infectious and did not want to interact with them” or “I tried to use protective gears most of the time). As a result, there was a noticeable reduction on the workload and how healthcare professionals (HCP) engaged with patients. For instance, one participant highlighted, “The attendance of outpatients decreased because patients were afraid of being isolated if they were suspected of having EVD.” Another participant shared, “I consistently found reasons to avoid going to work as I wanted to minimize my exposure.” Additionally, another individual remarked, “Whenever a patient exhibited bleeding from the nose or mouth, I had a strong suspicion that their condition might be critical, possibly leading to fatality.” In the realm of educational aspects, a participant expressed, “I gained valuable insights into EVD and expanded my understanding significantly.” Thus, although anxiety inducing and difficult, the outbreak increased awareness and knowledge about EVD. See Table 4.

### Feelings of HCPs toward announcement of another EVD outbreak

Participants expressed a diverse range of emotional responses for the potential challenges posed by another EVD outbreak in their work district. They expressed a heightened sense of anxiety and despair, anticipating feelings of fear, nervousness, terror, hopelessness, and perplexity if another EVD outbreak were to be announced in their district. For example, a participant said: “I would feel very nervous and terrified” and another stated “I would feel very hopeless and perplexed”. The sentiments expressed by participants, echoing those of others, indicated a profound level of fear, with some individuals expressing dissatisfaction with the country’s management of potential outbreaks. For example, one participant conveyed, “I would feel a great sense of disappointment in the way this country manages outbreaks.” Here, HCP felt someone disengaged from how their country’s officials were managing the outbreak, feeling somewhat unprotected from potential contamination.

A prevailing theme of resignation due to feelings of insecurity surfaced, as certain participants conveyed intense negative emotions when considering EVD and potential infection. Here, a participant said: “I would feel disgusted and I would just resign from work” or “I would feel unsafe and want to migrate to another district”, if infected. Thus, negative perspectives of EVD prevailed, creating concern and vulnerabilities among HCP. However, despite the mass inclination toward feelings of anxiety, despair, and resignation in the atmosphere, some participants conveyed a positive and proactive response. For example, one participant highlighted, “I would fight, train and support the community in the event of another EVD outbreak”. While some reported a sense of responsibility and commitment to collective action, other respondents expressed an indifferent attitude, as one participant said: “I would feel unbothered in the face of another potential outbreak”. See Table 4.

#### Reasons behind HCPs feelings toward recurrence of an EVD outbreak

The multifaceted reasons behind individuals’ feelings regarding the potential recurrence of an EVD outbreak included the complex interplay of various factors. Perceived severity of EVD was one of the reasons behind the feelings of anxiety and despair, which was largely directed toward the potential recurrence of an EVD outbreak. To illustrate, one participant noted, “EVD has a high rate of disease transmission, yet it kills so fast.” Another participant said, “Its management is unknown so it could erase mankind.” With the heightened concern about the lethality of the disease, apprehension regarding personal consequences also became evident— ‘hitting home’ for participants who feared for their safety and that of their loved ones if they were to be infected. For instance, a participant said, “I fear dying in a very scary way while bleeding from everywhere”. And another said “My greatest fear is acquiring the disease and I spread it to my family”.

Beyond the fears of personal consequences, concerns about preventive measures and preparedness emerged. A participant voiced: “People in Uganda hardly practice the necessary preventive precautions, so the disease will spread so fast”, another reinforced: “The country does not have what it takes to stop spread”. A third participant here echoed with concerns of how “There are no PPE at such times when they are most needed”. In the first except, the participant laments the lack of preventative measures to decrease risk of infection practiced by citizens, in the second excerpt, the sentiment is echoed as a failing of the country to contain EVD. The third participant’s words, however, reveal how the lack of PPE impacts their ability to protect themselves and each other, highlighting the dire need and demand that is unmet by supply.

Feelings of anxiety were exacerbated by past experiences and losses. For instance, one participant said, “Most of my relatives died due to EVD in the recent outbreak”. Another participant said “Health professionals usually die during EVD outbreaks and I am not any [one] special”. Such immediate experiences drive home the risk and make the lethality more real, removing opportunities to create distance between the HCP and the consequences of infection. Furthermore, participants acknowledged the heightened risks and concerns related to professional responsibilities. Here a participant said: “There is a high possibility of getting infected from work because of my profession which puts me at highest probability of getting in physical contact with infected persons”. Additionally, the profound impact on mental well-being was acknowledged was voiced, evidenced, for example in the words of a participant: “I would feel hopeless and not sure if I would live another day” or “I would be working under tension and fear and I would be overwhelmed all the time”.

Far reaching consequences extending beyond the professional realm to affect daily life and personal connections emerged as a factor contributing to negative emotions. Participants articulated these concerns with statements such as, “Another EVD could mean another lockdown, which ends up putting everything at a standstill,” and “Another EVD outbreak could cause the government to impose restrictions on movement and these would leave us confined and unable to see our loved ones or do business.” Thus, the effects of another EVD outbreak were viewed as possibly crippling for the country and citizens who would undergo extensive financial and personal hardship in response. See Table 4.

**Table 4:** Emergent themes and quotes under work experience during the recent EVD outbreak

Themes	Quotes
Themes under experience attending to general patients during the EVD outbreak	
Distress and Anxiety	• I felt like I would contract EVD anytime • I was very scared of every patient in case they later turned out to be having EVD • I was constantly stressed and overwhelming because being at work was unsafe • I was constantly exhausted from too much fear of contracting EVD from patients
Safety Measures and Protective Gear	• I considered every patient infectious and did not want to interact with them • I tried to use protective gears most of the time
Impact on Patient Interaction and Workload	• Turn up of OPD patients reduced as they feared to be isolated • Whenever a patient presented with bleeding from nose or mouth, I highly suspected they were going to die • I always got excuses not to work
Challenging and Educational Aspects	• I got to learn a lot about EVD
**Themes under feelings if another EVD outbreak was announced in the district of work**	
Anxiety and despair	• I would feel Scared and anxious • I would feel very nervous and terrified • I would feel very hopeless and perplexed • I would feel very disappointed in the country•
Resignation	• I would feel disgusted and I would just resign from work • I would feel unsafe and want to migrate to another district
Community support and action (Proactive response)	• I would feel ready to fight, train and support the community in the fight of the disease
Indifference	• I would feel unbothered
**Themes under reasons for feelings if another EVD outbreak was announced in the district of work**	
Perceived Severity of EVD	• Because EBV kills so fast and has a high rate of disease transmission • It’s management is not known yet its fatal • Another EVD outbreak could erase mankind
Fear of Personal Consequences	• I have fear of dying in a scary way while bleeding from EVD • I fear that I might acquire the disease and spreading it to my family
Concerns about Preventive Measures and Preparedness	• People in Uganda hardly practice the necessary preventive precautions, so the disease will spread so fast • The country does not have what it takes to stop spread • There are no PPE at such times when they are most needed
Impact on Daily Life and Work:	• Because of the possibility of another lockdown which would put everything at a stand still • There might be limitations in movement
Personal Experiences and Losses	• Most of my relatives died due to EVD • I lost friends to EVD in the last outbreak • Health professionals usually die during EVD outbreaks and I am not special
Impact on Mental and Emotional Well-being	• I would feel hopeless and not sure if I would live another day • I would be working under tension and fear and I would be overwhelmed all the time • I might lose loved ones
Professional Responsibility and Front-line Work	• There is a high possibility of getting infected from work because of my profession • I work in the emergency department, which is the entry point for most patients

## Discussion

In the current study, we assessed the presence of psychological distress (PD) among HCPs in Western Uganda following EVD outbreak and explored their experiences during the 2022 outbreak as well as their emotions and concerns toward announcement of another potential EVD outbreak. Our results highlighted diverse distressing emotional responses expressed by participants and provide insights into the complex experiences of HCPs during infectious disease outbreaks. The prevalence of psychological distress was 59.5% and being female, being a medical doctor and being a nurse/midwife increased the odds of psychological distress among HCPs.

The finding that more than half of the healthcare providers (HCPs) experienced psychological distress following the EVD outbreak is deeply concerning. This distress not only compromises the well-being of the affected individuals but also triggers concerns regarding the quality of care provided to patients [44]. This high prevalence aligns with the recurrent theme of expressed pervasive fear of contracting the virus while attending to patients. This theme is consistent with previous studies on outbreaks such as SARS, MERS, and EVD, our participants expressed significant psychological distress during the recent EVD outbreak [45]. Notably the prevalence was higher than that of 39.9% among HCPs in Saudi Arabia during the COVID-19 pandemic [46], likely because of the high death rate, quick spread, and horrifying symptoms associated with Ebola compared to COVID-19 [6, 7]. Additionally, the EVD outbreak occurred at a time when Uganda was still battling the COVID-19 pandemic [1]. The higher prevalence of psychological distress we found likely is related to differences in study tools used to assess psychological distress, the study setting, and the more supports available in Saudi Arabia.

Finding how being female increased the odds of psychological distress is likely associated with how most of the females HCPs were nurses/midwives who spend more time with patients in caregiving roles both in professional and personal contexts [47]. Therefore, the emotional toll of providing care during a disease outbreak, witnessing patient suffering, and grappling with the inability to save every patient may be more pronounced for female HCPs due to their occupational responsibilities and positioning [47]. This could also explain why being a nurse/midwife increased the odds of psychological distress compared to being a clinical officer; a category which comprised of only males in this study.

We found being a medical doctor compared to being a clinical officer, increased the odds of psychological distress, which illuminates the distinct difficulties faced by medical doctors throughout the outbreak [15–17]. Positioned on the frontlines of healthcare, doctors frequently shoulder significant duties, encompassing hands-on patient care, navigating high-stakes decisions under stress, and handling intricate medical scenarios [48]. This discovery may shed light on the emergent theme of safety explaining why HCPs adopted a cautious approach to safeguard themselves, such as viewing each patient as a potential carrier of EVD and refraining from physical contact. This reflects the profound impact on the perceived safety of healthcare settings that manifests with outbreaks [10]. The pressures intensified during the EVD outbreak, characterized by extended work hours, heightened risk of exposure to contagious illnesses, and firsthand exposure to patient suffering, all of which probably heightened their susceptibility to psychological strain [12, 48].

Our finding, that HCPs reported reduced workload due to the reduction in patient attendance, resulted from decreased healthcare-seeking behavior among citizens due to fear of isolation and suspicion of having EVD. A similar finding to that of the outbreak study in Sierra Leone [49], but contrary to a study in Germany where HCPs reported increased workload due to long shifts during EVD outbreak [12]. Differences in study findings are likely due to heightened fear, suspicion, a lack of public awareness on EVD symptoms, poor contact tracing, limited community involvement, and a lack of transport to facilities in African settings [50, 51], as well as increased health-system preparedness in Germany [52, 53]. A unique aspect of our findings is the reported increase in awareness and knowledge about EVD among HCPs, which echoes previous research revealing outbreaks can provide opportunities for learning and training [54].

HCPs described distressing feelings if another EVD outbreak was to occur—a source of worry and concern. Participants’ expressions of dissatisfaction with the nation’s handling of possible epidemics bear similarities to other studies [55, 56], indicating medical professionals may experience annoyance or disillusionment with the public health initiatives and the larger healthcare system during outbreaks. HCPs may voice discontent because of inadequate financing, inadequate infrastructure, and shortages of vital medical supplies, such as PPE, which make handling a subsequent EVD outbreak challenging [57]. HCPs may also become dissatisfied if they believe there is a lack of transparency, false information, or inadequate communication routes [58]. The prevalent theme of resignation stemming from feelings of insecurity implies vulnerability among HCPs. The sense of being unprotected from potential contamination resonates with broader literature on the myriad challenges faced by frontline workers, emphasizing the paramount importance of ensuring their safety and well-being [13].The perceived severity of EVD and fears of personal consequences, such as the prospect of a distressing demise and the potential spread of the disease to loved ones, align with previous studies highlighting the psychological impact of the perceived lethality of infectious diseases on HCPs [13, 14]. The references made by the participants to their personal EVD losses and past experiences help to build a more thorough knowledge of the long-lasting effects of epidemics on the mental health of medical professionals and on their lives. Experiences with personal loss have the power to exacerbate anxiety and create a stronger link with the possible outcomes of a new epidemic [27]. Recognizing the increased susceptibility to infection as a result of work obligations and voicing worries about psychological health highlight the complex issues facing HCPs [13]. The anticipations of extensive ramifications, such as the possibility of an additional lockdown and travel restrictions emphasize the wider societal and economic effects of infectious illness epidemics. These ramifications highlight the complex interactions occurring between a HCP’s personal and professional lives.

The results highlight the need for mental health interventions and programs for HCP support, particularly following public health emergencies such as epidemics of EVD. Providing psychological support services, putting stress management plans into place, and fostering a welcoming workplace that considers the particular requirements of HCPs, are a few possible strategies.

### Study strength and limitations

Our study is limited by potential recall bias, as our findings might have differed if data had been collected during, rather than eight months after, the EVD outbreak. Furthermore, certain variables that could enhance our understanding of factors associated with psychological distress, such as the duration of participants’ work experience, were not included in our assessment. Additionally, we acknowledge the possibility of social desirability bias impacting participant responses, which could influence the accuracy of our prevalence estimates. However, the mixed-methods design remains a significant strength of our study, providing both prevalence data on psychological distress and the contextual understanding behind these prevalence rates. Moreover, the use of multi-center sampling enhances the generalizability of our findings beyond a single setting.

## Conclusion

HCPs experience high levels of psychological distress in Mbarara district in Southwestern Uganda as a result of the 2022 EVD pandemic. Despite the outbreak’s terrifying effects on HCPs, the outbreak increased educational about the virus. HCPs expressed a wide range of feelings, such as dread, anxiety, despair, pessimism, and discontent with how the outbreaks are handled in Uganda. These findings emphasize the imperative to prioritize mental health support and interventions for this essential workforce, particularly in the context of potential future outbreaks.

## Data Availability

To protect study participant privacy, data are not publicly available but will be made available to appropriate academic parties on request from the corresponding author.

## References

[1] ECDC. Ebola virus disease outbreak in Uganda 2022. https://www.ecdc.europa.eu/en/ebola-virus-disease-outbreak-uganda.

[2] Ebola virus disease outbreak in Uganda. 2023. https://www.ecdc.europa.eu/en/ebola-virus-disease-outbreak-uganda#:~:text=Overall%2C%20nine%20Ugandan%20districts%20were,Masaka%2C%20Mubende%2C%20and%20Wakiso.

[3] Shoemaker T, MacNeil A, Balinandi S, Campbell S, Wamala JF, McMullan LK (2012). Reemerging Sudan Ebola virus disease in Uganda, 2011. Emerg Infect Dis.

[4] Iliyasu G, Ogoina D, Otu AA, Dayyab FM, Ebenso B, Otokpa D (2015). A multi-site knowledge attitude and practice survey of Ebola virus disease in Nigeria. PLoS ONE.

[5] MacNeil A, Farnon EC, Wamala J, Okware S, Cannon DL, Reed Z (2010). Proportion of deaths and clinical features in Bundibugyo Ebola virus infection, Uganda. Emerg Infect Dis.

[6] Vidal Y. How to prevent the spread of Ebola: effective strategies to Reduce Hospital Acquired infections. Lara Publications Inc; 2015.

[7] Goeijenbier M, Van Kampen J, Reusken C, Koopmans M, Van Gorp E (2014). Ebola virus disease: a review on epidemiology, symptoms, treatment and pathogenesis. Neth J Med.

[8] Do TS, Lee YS (2016). Modeling the spread of Ebola. Osong Public Health Res Perspect.

[9] Ridge LJ, Stimpfel AW, Dickson VV, Klar RT, Squires AP (2021). How clinicians manage routinely low supplies of personal protective equipment. Am J Infect Control.

[10] Spouse H. Classification of contacts and public health measures adopted for the secondary Ebola case, Madrid, 6 October–27 November 2014. Ebola virus disease. 2015:16.

[11] Chigwedere OC, Sadath A, Kabir Z, Arensman E (2021). The impact of epidemics and pandemics on the mental health of healthcare workers: a systematic review. Int J Environ Res Public Health.

[12] Lehmann M, Bruenahl CA, Löwe B, Addo MM, Schmiedel S, Lohse AW (2015). Ebola and psychological stress of health care professionals. Emerg Infect Dis.

[13] De Jong M, Reusken C, Horby P, Koopmans M, Bonten M, Chiche J (2014). Preparedness for admission of patients with suspected Ebola virus disease in European hospitals: a survey, August-September 2014. Eurosurveillance.

[14] Ji D, Ji Y-J, Duan X-Z, Li W-G, Sun Z-Q, Song X-A (2017). Prevalence of psychological symptoms among Ebola survivors and healthcare workers during the 2014–2015 Ebola outbreak in Sierra Leone: a cross-sectional study. Oncotarget.

[15] Rabelo I, Lee V, Fallah MP, Massaquoi M, Evlampidou I, Crestani R et al. Psychological distress among Ebola survivors discharged from an Ebola treatment unit in Monrovia, Liberia–a qualitative study. Front Public Health. 2016:142. 10.3389/fpubh.2016.00142 PMC4931229 27458576

[16] Chan M (2014). Ebola virus disease in West Africa—no early end to the outbreak. N Engl J Med.

[17] Jalloh MF, Li W, Bunnell RE, Ethier KA, O’Leary A, Hageman KM (2018). Impact of Ebola experiences and risk perceptions on mental health in Sierra Leone, July 2015. BMJ Global Health.

[18] Nejati V, Salehinejad MA, Sabayee A (2018). Impaired working memory updating affects memory for emotional and non-emotional materials the same way: evidence from post-traumatic stress disorder (PTSD). Cogn Process.

[19] Selvi Y, Özdemir PG, Özdemir O, Aydın A, Beşiroğlu L (2010). Influence of night shift work on psychologic state and quality of life in health workers. Dusunen Adam J Psychiatry Neurol Sci.

[20] Wald HS (2020). Optimizing resilience and wellbeing for healthcare professions trainees and healthcare professionals during public health crises–practical tips for an ‘integrative resilience’approach. Med Teach.

[21] Mosadeghrad AM (2014). Factors influencing healthcare service quality. Int J Health Policy Manage.

[22] Spoorthy MS, Pratapa SK, Mahant S (2020). Mental health problems faced by healthcare workers due to the COVID-19 pandemic–A review. Asian J Psychiatry.

[23] Stansfeld SA, Clark C, Caldwell T, Rodgers B, Power C (2008). Psychosocial work characteristics and anxiety and depressive disorders in midlife: the effects of prior psychological distress. Occup Environ Med.

[24] Mensah EA, Kisakye A, Gyasi SO. Ebola virus disease surveillance in the absence of a confirmed case; the case of the Rwenzori region of Uganda. Pan Afr Med J. 2020;37(1). 10.11604/pamj.2020.37.137.22957 PMC7757310 33425170

[25] Weyer J, Grobbelaar A, Blumberg L (2015). Ebola virus disease: history, epidemiology and outbreaks. Curr Infect Dis Rep.

[26] Bradfute SB, Jahrling PB, Kuhn JH. Ebola virus disease. Global virology I-identifying and investigating viral diseases. 2015:543 – 59.

[27] Raven J, Wurie H, Witter S (2018). Health workers’ experiences of coping with the Ebola epidemic in Sierra Leone’s health system: a qualitative study. BMC Health Serv Res.

[28] Dheda K, Perumal T, Moultrie H, Perumal R, Esmail A, Scott AJ et al. Tuberculosis in the time of COVID-19 1 The intersecting pandemics of tuberculosis and COVID-19: population-level and patient-level impact, clinical presentation, and corrective interventions. 2022. 10.1016/S2213-2600(22)00092-3 PMC8942481 35338841

[29] Kish L. Sampling organizations and groups of unequal sizes. American sociological review. 1965:564 – 72. 14325826

[30] Guest G, Bunce A, Johnson L (2006). How many interviews are enough? An experiment with data saturation and variability. Field Methods.

[31] Morse G, Salyers MP, Rollins AL, Monroe-DeVita M, Pfahler C (2012). Burnout in mental health services: a review of the problem and its remediation. Adm Policy Mental Health Mental Health Serv Res.

[32] Hu N, Li Y, He S-S, Wang L-L, Wei Y-Y, Yin L (2020). Impact of the family environment on the emotional state of medical staff during the COVID-19 outbreak: the mediating effect of self-efficacy. Front Psychol.

[33] Sanchez-Reilly S, Morrison LJ, Carey E, Bernacki R, O’Neill L, Kapo J (2013). Caring for oneself to care for others: physicians and their self-care. J Support Oncol.

[34] Purvanova RK, Muros JP (2010). Gender differences in burnout: a meta-analysis. J Vocat Behav.

[35] Shanafelt TD, Boone S, Tan L, Dyrbye LN, Sotile W, Satele D (2012). Burnout and satisfaction with work-life balance among US physicians relative to the general US population. Arch Intern Med.

[36] Bougie E, Arim RG, Kohen DE, Findlay LC (2016). Validation of the 10-item Kessler psychological distress scale (K10) in the 2012 Aboriginal peoples Survey. Health Rep.

[37] Andrews G, Slade T (2001). Interpreting scores on the Kessler psychological distress scale (K10). Aust N Z J Public Health.

[38] Kirabira J, Forry JB, Ssebuufu R, Akimana B, Nakawuki M, Anyayo L et al. Psychological distress and associated factors among hospital workers in Uganda during the COVID-19 lockdown–A multicentre study. Heliyon. 2022;8(1). 10.1016/j.heliyon.2022.e08807 PMC8769903 35075435

[39] Seruwagi G, Nakidde C, Lugada E, Ssematiko M, Ddamulira DP, Masaba A (2022). Psychological distress and social support among conflict refugees in urban, semi-rural and rural settlements in Uganda: burden and associations. Confl Health.

[40] Mutumba M, Resnicow K, Bauermeister JA, Harper GW, Musiime V, Snow RC (2015). Development of a psychosocial distress measure for Ugandan adolescents living with HIV. AIDS Behav.

[41] Vasiliadis H-M, Chudzinski V, Gontijo-Guerra S, Préville M (2015). Screening instruments for a population of older adults: the 10-item Kessler Psychological Distress Scale (K10) and the 7-item generalized anxiety disorder scale (GAD-7). Psychiatry Res.

[42] Plano Clark VL (2017). Mixed methods research. J Posit Psychol.

[43] Snodgrass JG, Clements KR, Nixon WC, Ortega C, Lauth S, Anderson M (2020). An iterative approach to qualitative data analysis: using theme, cultural models, and content analyses to discover and confirm a grounded theory of how gaming inculcates resilience. Field Methods.

[44] Palmer J, Ku M, Wang H, Crosse K, Bennett A, Lee E (2022). Public health emergency and psychological distress among healthcare workers: a scoping review. BMC Public Health.

[45] Chahley ER, Reel RM, Taylor S (2021). The lived experience of healthcare professionals working frontline during the 2003 SARS epidemic, 2009 H1N1 pandemic, 2012 MERS outbreak, and 2014 EVD epidemic: a qualitative systematic review. SSM-Qualitative Res Health.

[46] Al-Hanawi MK, Mwale ML, Alshareef N, Qattan AM, Angawi K, Almubark R et al. Psychological distress amongst health workers and the general public during the COVID-19 pandemic in Saudi Arabia. Risk management and healthcare policy. 2020:733 – 42. 10.2147/RMHP.S264037 PMC7354007 32753986

[47] Young KP, Kolcz DL, O’Sullivan DM, Ferrand J, Fried J, Robinson K (2021). Health care workers’ mental health and quality of life during COVID-19: results from a mid-pandemic, national survey. Psychiatric Serv.

[48] Migisha R, Ario AR, Kwesiga B, Bulage L, Kadobera D, Kabwama SN (2021). Risk perception and psychological state of healthcare workers in referral hospitals during the early phase of the COVID-19 pandemic, Uganda. BMC Psychol.

[49] James PB, Wardle J, Steel A, Adams J (2020). An assessment of Ebola-related stigma and its association with informal healthcare utilisation among Ebola survivors in Sierra Leone: a cross-sectional study. BMC Public Health.

[50] Akunzirwe R, Carter SE, Simbwa BN, Wanyana MW, Ahirirwe SR, Kizito SN et al. November. Time to Care and Factors Influencing Appropriate Sudan Virus Disease Care Among Case Patients in Uganda, September to 2022. 10.1016/j.ijid.2024.107073 38670481

[51] Granerud HS. The 2014–2016 Ebola Epidemic in West Africa. How did cultural factors contribute to an escalation of the outbreak? UiT Norges arktiske universitet; 2018.

[52] Kreitlow A, Steffens S, Jablonka A, Kuhlmann E (2021). Support for global health and pandemic preparedness in medical education in Germany: students as change agents. Int J Health Plann Manag.

[53] Blümel M, Busse R. The German health care system, 2015. International profiles of health care systems. 2015:69–76.

[54] Holding M, Ihekweazu C, Stuart JM, Oliver I (2019). Learning from the epidemiological response to the 2014/15 Ebola virus disease outbreak. J Epidemiol Global Health.

[55] Willis K, Ezer P, Lewis S, Bismark M, Smallwood N (2021). Covid just amplified the cracks of the system: working as a frontline health worker during the COVID-19 pandemic. Int J Environ Res Public Health.

[56] Pieterse P, Lodge T (2015). When free healthcare is not free. Corruption and mistrust in Sierra Leone’s primary healthcare system immediately prior to the Ebola outbreak. Int Health.

[57] Kibuule M, Sekimpi D, Agaba A, Halage AA, Jonga M, Manirakiza L (2021). Preparedness of health care systems for Ebola outbreak response in Kasese and Rubirizi districts, Western Uganda. BMC Public Health.

[58] Kutalek R, Klocker RI. Community engagement in clinical trials and the implementation of experimental Ebola vaccines in Sub-saharan Africa–A scoping review.

